# Pyruvate Kinase M1/2 Proteoformics for Accurate Insights into Energy Metabolism Abnormity to Promote the Overall Management of Ovarian Cancer Towards Predictive, Preventive, and Personalized Medicine Approaches

**DOI:** 10.3390/metabo15030203

**Published:** 2025-03-16

**Authors:** Yan Wang, Nuo Xu, Marie Louise Ndzie Noah, Liang Chen, Xianquan Zhan

**Affiliations:** 1 Department of Gynecological Oncology, Shandong Cancer Institute, Shandong First Medical University & Shandong Academy of Medical Sciences, 440 Jiyan Road, Jinan 250117, China; ywang@email.sdfmu.edu.cn (Y.W.); nxu@email.sdfmu.edu.cn (N.X.); mlnn@email.sdfmu.edu.cn (M.L.N.N.); 2 Shandong Provincial Key Laboratory of Precision Oncology, Shandong Cancer Institute, Shandong First Medical University & Shandong Academy of Medical Sciences, 440 Jiyan Road, Jinan 250117, China; 3 Department of Gynecology, Gaotang County Medical Center, Liaocheng 252800, China; 4 Shandong Provincial Key Medical and Health Laboratory of Ovarian Cancer Multiomics & Jinan Key Laboratory of Cancer Multiomics, Medical Science and Technology Innovation Center, Shandong First Medical University, 6699 Qingdao Road, Jinan 250117, China

**Keywords:** ovarian cancer, pyruvate kinase M1/2, proteoform, proteoformics, pyruvate kinase M1/2 proteoforms, energy metabolism, post-translational modification, therapeutic target, biomarker, predictive preventive personalized medicine (PPPM/3PM)

## Abstract

Ovarian cancer (OC) is a global health problem that frequently presents at advanced stages, is predisposed to recurrence, readily develops resistance to platinum-based drugs, and has a low survival rate. Predictive, preventive, and personalized medicine (PPPM/3PM) offers an integrated solution with the use of genetic, proteomic, and metabolic biomarkers to identify high-risk individuals for early detection. Metabolic reprogramming is one of the key strategies employed by tumor cells to adapt to the microenvironment and support unlimited proliferation. Pyruvate kinases M1 and M2 (PKM1/2) are encoded by the *PKM* gene, a pivotal enzyme in the last step of the glycolytic pathway, which is at the crossroads of aerobic oxidation and the Warburg effect to serve as a potential regulator of glucose metabolism and influence cellular energy production and metabolic reprogramming. Commonly, the ratio of PKM1-to-PKM2 is changed in tumors compared to normal controls, and PKM2 is highly expressed in OC to induce a high glycolysis rate and participate in the malignant invasion and metastatic characteristics of cancer cells with epithelial/mesenchymal transition (EMT). PKM2 inhibitors suppress the migration and growth of OC cells by interfering with the Warburg effect. Proteoforms are the final structural and functional forms of a gene/protein, and the canonical protein PKM contains all proteoforms encoded by the same *PKM* gene. The complexity of PKM can be elucidated by proteoformics. The OC-specific PKM proteoform might represent a specific target for therapeutic interventions against OC. In the framework of PPPM/3PM, the OC-specific PKM proteoform might be the early warning and prognosis biomarker. It is important to clarify the molecular mechanisms of PKM proteoforms in cancer metabolism. This review analyzes the expression, function, and molecular mechanisms of PKM proteoforms in OC, which help identify specific biomarkers for OC.

## 1. Introduction

Ovarian cancer (OC) is a global disease that is often diagnosed at an advanced stage, without effective screening strategies. Each year, 324,398 new cases of OC are diagnosed worldwide, and 206,839 of these cases result in cancer deaths [1,2]. OC is subdivided into at least five histological subtypes, of which epithelial OC (EOC) accounts for ~90% of OCs. EOC includes endometrioid, serous, mucinous, and clear cell carcinomas, of which low-differentiation high-grade serous carcinoma (HGSC) is the predominant and highly aggressive subtype. Other rare histological types include small cell carcinomas of uncertain histological origin and carcinosarcomas. Non-EOC accounts for approximately 10% of OCs, including sex cord-stromal tumors and germ cell tumors. The first-line treatment regimen for OC involves (i) performing primary cytoreductive surgery, followed by platinum-based chemotherapy, or (ii) administering neoadjuvant chemotherapy (preoperative chemotherapy), followed by interval debulking surgery and subsequent postoperative adjuvant chemotherapy [2]. OC is characterized by a high propensity for recurrence and escalating resistance to chemotherapy, eventually leading to the development of platinum-based drug resistance, thereby resulting in poor survival rates. The 5-year survival rate of HGSC is as low as 30%, underscoring the formidable challenge in the management of this disease and emphasizing the need to devise novel therapeutic approaches [3,4]. The predictive, preventive, and personalized medicine (PPPM) model offers a comprehensive solution by integrating predictive tools to identify high-risk populations through genetic, proteomic, and metabolic biomarkers, enabling early detection. Preventive strategies, tailored based on individual risk profiles, can significantly reduce incidence. The personalized aspect of PPPM ensures that treatments are adapted to the molecular characteristics of each patient’s cancer, improving response rates and overcoming platinum resistance. By combining early diagnostic markers, preventive interventions, and personalized therapies, the PPPM model addresses the complex challenges of OC, paving the way for more effective and individualized management of the disease [5,6,7].

Over the past few decades, more than 100 protein biomarkers have been developed for clinical diagnosis, and many of them have been approved by the US Food and Drug Administration (FDA). Human epididymis protein 4 (HE4) and carbohydrate antigen 125 (CA125) are the most widely used protein biomarkers for diagnosing OC, treatment monitoring, and disease recurrence evaluation. However, most protein biomarkers face an undisputable dilemma due to insufficient specificity and/or sensitivity [8,9]. Therefore, within the framework of PPPM, it is essential to identify efficient, accurate, and highly specific protein biomarkers as OC diagnostic criteria, therapeutic targets, and new molecules for prognosis.

In tumor cells, metabolic reprogramming is a key strategy employed to adapt to the microenvironment and support unlimited proliferation [10]. The most famous example is the Warburg effect, which refers to the tendency of cancer cells to generate energy through glycolysis rather than the more efficient oxidative phosphorylation (OXPHOS), even though oxygen is sufficient. This results in the production of large amounts of lactate. The intermediate products generated by glycolysis can provide cells with the necessary materials for biosynthesis, such as nucleotides, amino acids, and lipids, supporting the rapid proliferation of tumor cells. A review article summarized a scholar’s perspective that pancreatic cancer cells undergo metabolic reprogramming, transitioning from OXPHOS to glycolysis. This transformation is considered capable of regulating cascading reactions associated with invasion and metastasis while also promoting EMT, tumor angiogenesis, and distant metastasis [11].

Recently, an increasing focus has been put on the role of glycolysis in OC [12]. Glycolysis can affect prognosis by promoting cancer cell proliferation, invasion, metastasis, and chemotherapy resistance and is involved in multiple stages of OC development [13,14,15]. Lactic acid is the final product of glycolysis and serves as an immunomodulatory compound in the tumor microenvironment (TME). It can be absorbed by endothelial cells and associated fibroblasts [16,17], triggering the production of various cytokines and vascular endothelial growth factors. This promotes OC invasion and metastasis, whereas excessive lactic acid production leads to TME acidification, creating an immunosuppressive microenvironment that is a key mechanism for cellular immune escape. Within the TME, lactate can induce a phenotypic shift of tumor-associated macrophages (TAM) from M1 to M2, thereby facilitating tumor progression [18]. Specifically, M1-TAMs are characterized by their pro-inflammatory phenotype, which relies on glycolysis to inhibit cancer cell proliferation. Conversely, M2-TAMs exhibit an anti-inflammatory phenotype and promote cancer cell proliferation and metastasis via OXPHOS.

**Figure 1:**
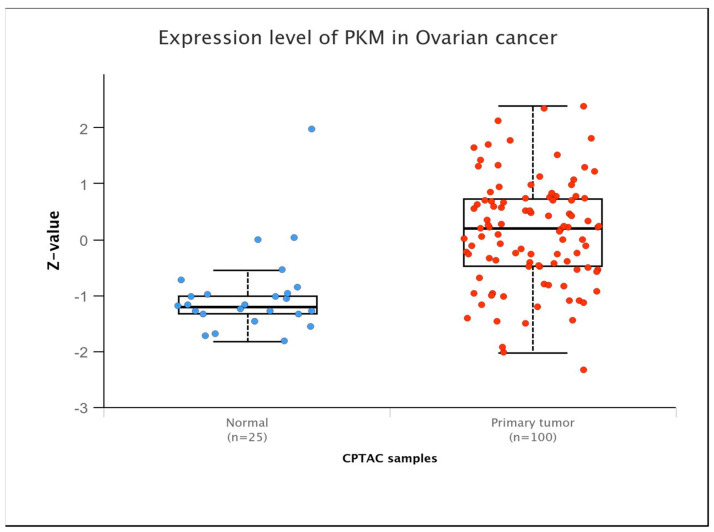
PKM expression levels in ovarian cancers at the protein level (Ualcan database).

Glycolysis regulation is contingent upon the activities of three pivotal enzymes: hexokinase, 6-phosphofructokinase-1, and pyruvate kinase (PK). PK is the key enzyme controlling the final step of the glycolytic process, helping to convert phosphoenolpyruvate (PEP) into pyruvate while also producing one molecule of ATP. The final step lies at the intersection of aerobic oxidation and the Warburg effect, positioning PK as a potential regulator of glucose metabolism. PK exists in four isozymes: L, R, M1, and M2. PKL and PKR are encoded by the *PKLR* gene, and PKM1 and PKM2 are encoded by the *PKM* gene. These isozymes exhibit tissue-specific expression patterns and possess distinct physiological functions and regulatory mechanisms. Studies found that PKM was overexpressed in OC at the protein level, particularly in its advanced stage (Figure 1). Studies have demonstrated that upregulation of PKM2 induces enhanced glycolysis rates in OC cells, while inhibition of PKM2 with specific inhibitors hampers the migration and proliferation of these cells by interfering with the Warburg effect [19]. Quantitative proteomic analysis of OC tissues identified 205 differentially expressed proteins, of which PKM2 exhibited significantly increased protein expression in OC [20]. Comprehensive analysis of transcriptomic data from The Cancer Genome Atlas (TCGA) database also demonstrated that PKM2 has significantly elevated transcript levels in many cancers, including OC, especially in highly invasive tumor phenotypes [21]. Moreover, AKT2 upregulates the expression of STAT3 and activates NF-κB via PKM2, thereby augmenting the motility and invasive capacities of OC cells in vitro [22]. PKM2 phosphorylates histone H3, aiding gene expression and driving tumor development [23].

The chemical essence of PKM is a protein, and its activity is regulated by PTMs, allosteric effectors, and hormonal signaling pathways, which subsequently affect the proliferation, invasion, and angiogenesis of tumor cells. Consequently, it has potential as a protein biomarker for OC and represents an attractive therapeutic target. Proteoforms focus on the structural and functional changes in a protein [24]. PKM proteoform can provide insights into the intricate nature of PKM and may serve as a potential target for therapeutic interventions against diseases and as biomarkers for accurate prediction or prognosis in the framework of PPPM. This article proposes the utilization of proteoformics to investigate the role and regulatory mechanisms of PKM proteoforms in OC, facilitate PPPM, and provide insights into the search for biomarkers of this global cancer. Additionally, this review is a narrative synthesis of the literature based on the authors’ expertise in tumor metabolism and PKM. Studies were selected based on relevance to the topic and their contribution to the understanding of PKM in the tumor microenvironment.

## 2. Proteoform and Proteoformics

The execution of genome functions primarily occurs at the protein level, with the diversity of biological functions determined by different protein types. This diversity arises from a combination of allelic differences at the DNA level, alternative splicing in RNA transcripts, epigenetic changes affecting both DNA and RNA, and a series of PTMs at the protein level, which are called proteoforms, to play an active role in numerous biological processes, such as regulating gene expression, energy metabolism, and cell signaling. The concept of proteoforms provides an in-depth understanding of the proteome and underscores the inherent complexity of proteins [25].

**Figure 2:**
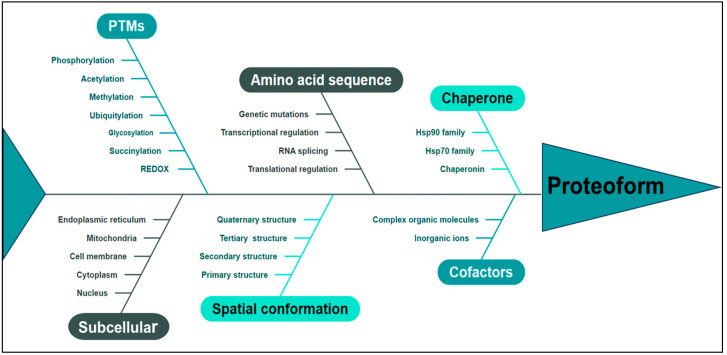
The proteoform is determined by multiple pivotal factors, including amino acid sequence, PTMs, spatial conformation, cofactors, chaperone, subcellular localization, and functional attributes. PTMs: post-translational modifications.

Proteoforms represent the ultimate structural and functional manifestation of a gene or protein, serving as the fundamental building block of the proteome and embodying an advanced level of investigation in proteomics, thereby delineating the future trajectory of protein research. In 2023, the term “Proteoformics” was introduced [26], which is the theory and methodology of investigating proteoforms. The primary aim of proteoformics is to comprehensively explore the heterogeneous nature of proteoforms and their dynamic alterations across cellular, tissue-specific, organ-specific, and organismal contexts and elucidate the functional implications of these proteoforms in both normal physiology and disease pathogenesis in human biology. Proteoformics employs high-throughput analytical methodologies and technologies to identify, characterize, and quantitatively assess diverse proteoforms and their respective functionalities. This study unveils a paradigm shift in the comprehension of two-dimensional gel electrophoresis (2DGE) by demonstrating that every 2D gel spot contains numerous proteoforms, ranging from dozens to hundreds or even thousands [27]. This discovery challenges the conventional notion held for over 40 years, which posits that each spot solely represents 1–2 proteins. Integrating 2DGE with liquid chromatography/mass spectrometry (2DE-LC/MS) forms a high-throughput technological platform to effectively separate and identify human proteoforms, thereby providing indispensable technical support for large-scale investigations into proteoforms [27]. The advancement of 2DE-MS has facilitated identifying large-scale proteoforms, thereby enhancing the conceptual framework of proteoform characterization [28,29]. The pivotal factors to determine a proteoform [24,30,31] are shown in Figure 2. Utilizing MS-based proteomics data for gene annotation improvement, proteogenomic analysis was conducted on 242 cases of HGSC, which identified a biomarker comprising 64 proteins with high specificity in predicting resistance to initial platinum-based drug treatment in a subset of HGSC patients [32]. The current study utilized quantitative proteomics to comprehensively profile diverse histological subtypes of EOC. This approach was used to analyze protein expression patterns, which identified a set of proteins with dysregulated expression in EOC tissues. Our findings shed light on the molecular features and potential therapeutic targets of different histological subtypes [33]. Furthermore, this study identified aberrant signaling pathways associated with cancer, including the KEGG pathway, DNA replication, cell cycle, HIF-1 signaling pathway, and several metabolism-related pathways exhibiting excessive protein expression. These findings underscore the pivotal role of these signaling pathways in the initiation and progression of EOC [33]. PKM is widely involved in the above abnormal pathways. PKM2 directly interacts with the HIF-1α subunit, augmenting its binding to hypoxia response elements through interaction with prolyl hydroxylase 3 (PHD3). This interaction enhances the activation of HIF-1 target genes by recruiting the coactivator p300 acetyltransferase, thereby establishing a positive feedback loop for reprogramming glucose metabolism in cancer cells [34].

Proteoformics research is involved in the following areas. (i) Identification and quantification of proteoforms: High-throughput methods are used to identify and measure various proteoforms encoded by the same gene as well as their respective abundances [28,29,35,36]. (ii) PTMs: the identification of proteoform covalent modifications, such as phosphorylation, acetylation, methylation, and ubiquitination, are achieved through the use of high-throughput and highly sensitive methodologies that include enzymatic or chemical reactions [35,36,37]. (iii) Protein structure analysis: By integrating advanced techniques such as MS, nuclear magnetic resonance spectroscopy (NMR), and Alpha-Fold3, one can comprehensively investigate the structural characteristics of diverse proteoforms to gain insight into their functional attributes, intricate interactions, and underlying mechanisms of action [29,35]. (iv) Functional studies of proteoforms: Functional assays, such as the determination of enzyme activity and detection of protein/protein interactions, are employed to investigate the functionality and interplay among distinct proteoforms. These assays reveal the protein network and explore the impact of diverse proteoforms on biological functions and phenotypes at cellular, tissue, organ, and organismal levels [33,35,38]. (v) The localization and dynamics of proteoforms: Using fluorescence microscopy and live-cell imaging, proteins are labeled to study their localization and dynamic changes within cells, revealing the functions and positional variations of proteins during cellular processes [35]. (vi) Data analysis: Establish and maintain a robust protein database, develop and employ advanced bioinformatics and computational tools to analyze and interpret extensive proteoform datasets, and integrate multi-omics data (such as genomics, transcriptomics, and metabolomics) for pathway analysis and network construction to facilitate comprehensive systems biology analysis and elucidate the intricate nature of proteoforms in biological systems [33,39].

**Figure 3:**
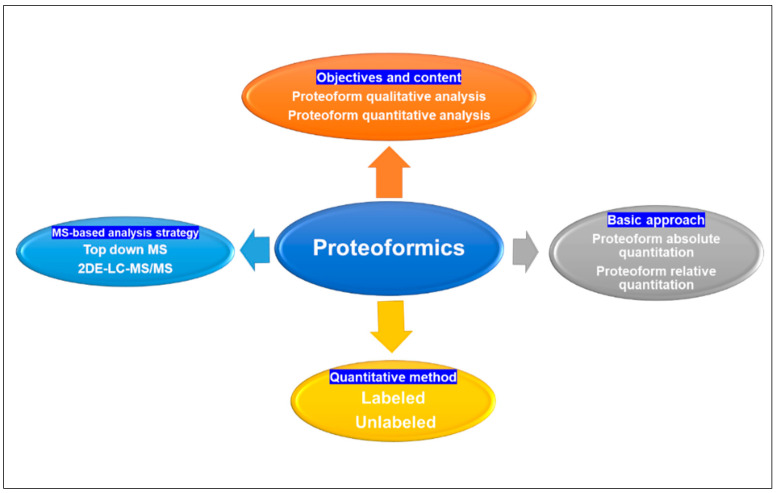
Proteoformics contains qualitative, quantitative, and functional analysis of proteoforms; quantification analysis of proteins can be categorized into absolute and relative quantification basic approaches, and the quantification can be achieved by utilizing labeled and unlabeled methods. Proteoformics employed the MS-based analytical strategies top-down MS and 2DE-LC/MS to investigate proteoforms. MS: mass spectrometry. 2DE: two-dimensional gel electrophoresis. LC: liquid chromatography.

From an analytical chemistry perspective, proteoformics is divided into qualitative, quantitative, and functional analyses of proteoforms within a group of proteins. Qualitative analysis entails the identification of proteoform sequences, proteoform/proteoform interactions, and PTMs, whereas quantitative analysis focuses on comparing alterations in proteome-wide protein expression levels under distinct physiological conditions to discern proteins with regulatory functions and gain further insights into their association with disease mechanisms [40]. Quantitative analysis can be categorized into absolute and relative quantification methods [40]. Absolute quantification analysis was used to determine the absolute amount of proteoforms (mass, molar, or copy number) in each sample. Absolute quantification is usually performed at the peptide level and requires higher resolution and sensitivity on the mass spectrometer. Labeled absolute quantification (AQUA) [41] is the most widely used, unlabeled estimation of the absolute quantity of a proteoform by comparing its signal intensity in a sample with low accuracy. Currently, commonly used quantitative methodologies have 2DGE [27,42] for separation and quantification or the integration of LC-MS [43,44]. The quantification of 2DGE can be achieved by utilizing DIGE labeling and non-labeled 2DE techniques. LC-MS enables quantification through labeled ICAT [45,46], iTRAQ [47,48], TMT [49,50], SILAC [51], enzyme labeling methods, as well as non-labeled label-free [52,53] approaches (Figure 3).

The human proteome is estimated to have at least one million and even up to one billion distinct proteoforms. It is imperative to systematically investigate, discern, and quantify the extensive array of proteoforms on a large scale. Currently, two primary methodologies are employed for the investigation of proteoforms: top-down MS and 2DE-LC/MS, each of which has advantages and disadvantages in analyzing the proteoforms [30].

(i) Top-down MS: Direct analysis of intact and undigested proteoform molecules. Initially, proteoform separation techniques such as capillary zone electrophoresis (CZE) and LC are used to isolate proteoforms. The isolated proteoforms are then subjected to MS/MS analysis. Finally, the amino acid sequence and PTMs are identified with MS/MS data and protein database [54,55]. The drawback of this approach lies in its limited analysis throughput caused by ion suppression effects, rendering it suitable primarily for proteoforms with a molecular weight below 30 kDa, where direct analysis of proteoforms in complex samples, such as whole cell lysates, may result in overlapping signals that are difficult to resolve.

(ii) 2DE-LC/MS: First, the proteoform samples are separated with 2DE based on their isoelectric point (p*I*) and relative mass (*M_r_*). The separated proteoforms are digested into peptide fragments with trypsin and identified by MS/MS. Finally, the amino acid sequences and PTMs obtained from the MS/MS data are matched in a protein database [27,30]. Due to the presence of a diverse range of proteoforms, ranging from fifty to several hundred, within each 2D-gel spot and the ability of 2DE to effectively separate low-abundance proteoforms, the utilization of 2DE-LC/MS holds immense potential for high-throughput analysis to determine the existence of various proteoforms [27].

In the field of proteoformics, particularly when investigating proteoforms associated with a specific gene or protein, it is imperative to carefully select appropriate antibodies that specifically target this protein. This antibody can be utilized for the purification of a specific protein intended for top-down MS analysis or for conducting 2DE/Western blotting (2DE-WB) to identify distinct proteoforms of the target protein or gene, followed by MS analysis employing the positively identified immunoblotting proteoform [56,57]. In top-down MS analysis, stable isotope labeling enables quantitative assessment of different proteoforms of each specific protein or gene in both the target and control groups.

In the context of non-targeted proteoformics research, it is crucial to accurately detect and quantify distinct variations between the target and comparison groups. For instance, stable isotope labeling techniques, such as TMT, are employed to label distinct proteoforms in both the target and control groups, followed by thorough mixing of TMT-labelled samples, which can be utilized for top-down MS or 2DE-LC/MS analyses, enabling the identification and quantification of distinct proteoforms between target and control cohorts [31,50].

Proteoformics offers a novel perspective for the identification of disease-related biomarkers and potential molecular targets. Investigating OC with proteoformics can provide a more comprehensive understanding, tumor stratification, predictive diagnosis, prognosis assessment, and personalized medical services under the framework of PPPM and enhance the understanding of malignant transformation and treatment strategies.

## 3. Working Hypothesis in the Framework of 3P Medicine

We hypothesize that PKM proteoforms differ significantly between OCs and normal controls, early and advanced OCs, platinum-resistant and platinum-sensitive OCs, metastatic and primary OCs, and various histological types. These differential PKM proteoforms may be crucial for the metabolic regulation of OC cells, serving as potential therapeutic targets and biomarkers for accurate prediction and prognosis within the 3PM framework. This work offers valuable insights into OC development, platinum resistance, PKM proteoform biomarkers, and the realization of 3PM.

## 4. PKM Proteoformics

### 4.1. Alternative Splicing of RNA Transcripts Encoding PKM

RNA splicing is a pivotal process in the transcriptional regulation of the majority of human genes. Dysregulated RNA splicing is a ubiquitous molecular characteristic of diverse tumor types. Cancer-associated dysregulation of splicing can facilitate tumorigenesis through a multitude of mechanisms, including augmented cellular proliferation, diminished apoptotic activity, heightened migratory and metastatic potential, increased resistance to chemotherapy, and evasion of immune surveillance [58,59]. For four PK isoenzymes, L, R, M1, and M2, PKL is mainly expressed in the pancreas, liver, intestines, and kidneys. PKR is predominantly expressed in red blood cells [60], PKM1 is mainly expressed in high energy-consuming tissues such as the heart, brain, and skeletal muscles, and PKM2 is mainly expressed in high-proliferation cells and high synthetic metabolic cells, such as tumor cells, embryonic cells, and stem cells [61,62,63,64,65]. PKM1 and PKM2 are two splicing variants of the *PKM* gene, and this alternative gene splicing can dysregulate cellular metabolism.

Exons 9 and 10 of the *PKM* gene exhibit mutually exclusive splicing events during pre-mRNA processing, leading to alternative splicing variants of PKM. The *PKM1* mRNA harbors the 9th exon, whereas the *PKM2* mRNA harbors the 10th exon, leading to the generation of two variants, namely PKM1 and PKM2 [61]. Despite catalyzing the same reaction, PKM1 and PKM2 exhibit distinct properties, including oligomerization, enzymatic activity, and regulatory mechanisms, which can be attributed to a mere 22 amino acid differences [61]. The presence of the 9th exon in *PKM1* enables it to maintain persistent enzyme activity as a tetramer. In contrast, exon 10 regulates PKM2 activity, allowing for a switch between active and inactive states to maintain a balance between energy utilization and synthesis demands based on nutrient availability [62,63,64,65]. Elevated PKM2 levels in human tumors have been correlated with reduced patient survival, advanced disease stage, and unfavorable prognosis [61]. The ubiquitin ligase KBTBD8 modulates the abundance of PKM1 via the Erk1/2 and Aurora A signaling pathways [66]. However, the regulation of these isoforms extends beyond transcription. Post-transcriptional regulation mechanisms, including splicing, translation, and protein modifications, further affect the final protein products and their functional properties.

Alternative splicing of *PKM2* is regulated through interactions with PTBP1, hnRNPA1, or hnRNPA2 to suppress exon 9 inclusion or with SRSF3 to enhance exon 10 inclusion [63,64,65,67,68]. Moreover, the use of ASO small nucleic acid drugs to modulate the splicing pattern of *PKM* pre-mRNA and facilitate the production of PKM1 instead of PKM2 in cancer cells can effectively impede cell proliferation and delay tumor formation. These findings suggest that PKM1 has the potential to suppress tumor activity. Consequently, these findings suggest a promising therapeutic strategy for cancer treatment that downregulates PKM2 expression and upregulates PKM1 expression in cancer cells [69]. Transcription factor 9 (LHX9) enhances the activity of PKM2 by directly binding to its promoter region, thereby inducing metabolic reprogramming of glycolysis in cancer cells and promoting their malignant characteristics. LHX9 knockdown inhibits PKM2 activity and reprograms glycolysis, resulting in decreased tumorigenicity in nude mice [70].

### 4.2. Structural Analysis of PKM

The PK isoenzyme is about 250 KD, maintains as a tetramer with 4 subunits, and each subunit is about 55 KD [60], with different physiological and pathological functions. The liver-specific PKL plays a pivotal role in regulating blood glucose levels through its involvement in glycolysis and gluconeogenesis, modulating both pyruvate production and breakdown. Erythrocyte-specific PKR facilitates the provision of energy for the maintenance of the normal functionality and lifespan of these cells. PKM1 possesses a stable tetrameric structure comprising four identical subunits, which facilitates glycolysis to generate ATP to support high-intensity and sustained energy demand. PKM2 exhibits structural variations in tetrameric, dimeric, and monomeric forms, allowing for dynamic interconversion between these states [71,72]. The tetrameric PKM2 proteoforms exhibit high catalytic activity and mainly participate in metabolic reactions, promoting the conversion of phosphoenolpyruvate (PEP) to pyruvate during glycolysis in normal cells. The dimeric PKM2 proteoforms have lower catalytic activity and play a role in transcriptional regulation within the cell nucleus, which is involved in cellular metabolic reprogramming, enhancing the Warburg effect [73] to provide the metabolites necessary for rapid cancer cell growth [74]. Its translation is regulated by PTMs such as phosphorylation, acetylation, and ubiquitination, as well as by metabolites like fructose-1,6-bisphosphate (Fru-1,6-BP) [75,76].

The dimerization proteoform of PKM2 refers to the process by which two PKM2 molecules undergo non-covalent interactions, resulting in conformational changes in protein tertiary and quaternary structures. The dimeric proteoform of PKM2 is capable of translocating into the cell nucleus and acting as a transcriptional coactivator, thereby regulating gene expression and actively participating in cellular proliferation and signal transduction [59]. An OC cell line was established, and LC-MS/MS analysis was conducted on the cells. Expanding upon the ECAR experiments, they further investigated the impact of the ESM1-PKM2 axis on cellular glucose metabolism. The results indicated that ESM1 plays a crucial intermediary role between PKM2 and UBA2, promoting PKM2 methylation and dimerization, thereby enhancing the Warburg effect and facilitating PKM2 nuclear translocation. Ultimately, this leads to the phosphorylation of STAT3, strengthening both the OC glycolysis pathway and vasculogenic mimicry (VM) [77]. The study also revealed that shikonin (SHK), a natural compound with anti-cancer properties, effectively impedes the molecular interaction between ESM1 and PKM2, thereby inhibiting the formation of PKM dimers and subsequently suppressing glycolysis, fatty acid synthesis, and angiogenesis in OC [77].

Cofactors trigger structural modifications in PKM2, and its oligomeric state is modulated by various endogenous allosteric regulators in vivo. Fru-1,6-BP, an activator of PKM2 conformational change, stabilizes the tetrameric form of PKM2 and converts its inactive T state to a fully active R state [78]. The two conformational transitions exhibit a “seesaw effect” mechanism, indicating the significance of intermolecular interactions at the A–A’ or C–C’ interfaces in these two PKM2 conformational changes [79]. Serine facilitates tetramerization of PKM2, thereby increasing its enzymatic activity [80]. Analysis of PKM2 and the serine complex crystal (PDB: 5X0I) revealed that serine can bind to an alternative pocket site instead of the Fru-1,6-BP binding site. The “variable resistor” mechanism is used to regulate PKM2 by serine. In other words, when serine is abundant, it can activate tetramerization of PKM2 to support aerobic glycolysis to produce lactate. However, when serine is deficient, it reduces the activity of PKM2 and redirects glucose-derived carbons towards the serine synthesis pathway to promote serine synthesis. This mechanism is crucial for the growth and survival of cancer cells [81]. Succinyl aminoimidazole carboxamide ribose-5′-phosphate (SAICAR) can trigger the dimeric proteoform of PKM2 [82]. The direct binding of SAICAR to PKM2 enhances the affinity of PKM2 for its substrate PEP, increases catalytic turnover, and improves cell survival under glucose-deprived conditions. In PKM2, the substitution of glutamine with arginine at position 393 renders PKM2 insensitive to SAICAR, but this mutant can still be activated by Fru-1,6-BP. This suggests that the binding site of SAICAR on PKM2 is not located within the Fru-1,6-BP binding pocket [83]. SAICAR also induces the nuclear translocation of PKM2 and enhances its protein kinase function. The complex of PKM2 with SAICAR can phosphorylate many proteins for cell proliferation, which is essential for maintaining the signaling pathway of tumor cell growth [84]. Triiodothyronine (T3) interacts with PKM2 to stabilize it in an inactive monomeric form and inhibit its enzymatic activities. T3 also suppresses the assembly of PKM2 tetramers, which promote cell proliferation. However, high concentrations of Fru-1,6-BP can counteract the inhibitory effects of T3 [85].

### 4.3. Different Subcellular Localizations of PKM2 Promote Tumor Growth

#### 4.3.1. Nuclear Translocation

**Figure 4:**
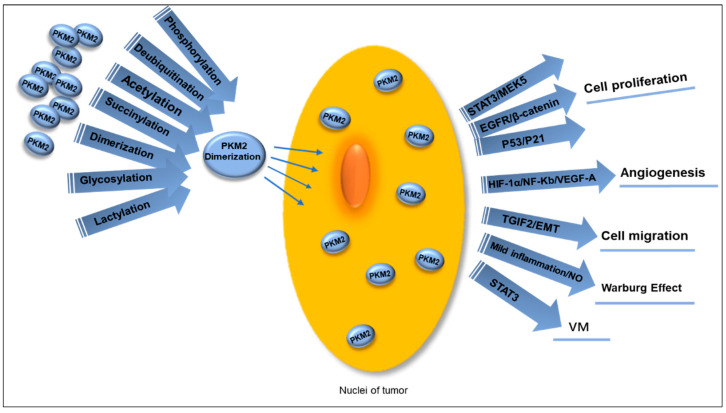
Multiple PTMs guide PKM2 dimerization, promoting its translocation into the nucleus to function as a transcription coactivator in various ways. This process facilitates tumor cell proliferation through the STAT3/MEK5, EGFR/β-catenin, and P53/P21 pathways, activates VEGF-A and stimulates tumor angiogenesis by interacting with NF-Kb and HIF-1α, and leads to EMT and promotes tumor cell migration when bound to TGIF2. Mild inflammatory stimulation generates NO, which contributes to the occurrence of the Warburg effect. Simultaneously, nuclear PKM2 induces phosphorylation of STAT3 to enhance VM in OC. PTMs: post-translational modifications. PKM2: pyruvate kinases M2. EMT: mesenchymal transition. NO: nitric oxide. VM: vimentin. OC: ovarian cancer.

PKM2 can enter the nucleus through multiple pathways, and the dimeric proteoform can undergo nuclear translocation (Figure 4). One of the key roles of nuclear PKM2 (nPKM2) is to act as a transcriptional coactivator, regulating the expression of numerous genes linked to tumor development and progression. The expression of key proteins such as Oct4, β-catenin, HIF-1α, and STAT3 is regulated by nPKM2. The protein kinase activity of nPKM2 can be exerted either as a dimer or a monomer. PKM2 protein kinase exhibits dual specificity, functioning as a tyrosine kinase and serine/threonine kinase. This emphasizes the multifunctionality of protein kinase activity and its various regulatory roles. nPKM2phosphorylates the residue Tyr105 in STAT3, thereby activating the transcription of MEK5 to participate in cell proliferation [86] and also interacting with NF-κB and HIF-1α in the cell nucleus to activate the VEGF-A expression, thereby stimulating tumor angiogenesis [87]. The transactivation of PKM2-dependent β-catenin is crucial for EGFR-driven cancer cell proliferation. PKM2 binds to the phosphorylated β-catenin at residue pTyr333 to form a complex, which is recruited to the CCND1 promoter, resulting in the expression of cyclin D1 through acetylation of H3 and elimination of HDAC3 [88]. The PKM2 protein also inhibits the p53 protein (a tumor suppressor gene) and prevents the P21 gene activation, resulting in sustained G1 phase of tumor cells to promote the ongoing cancer cell proliferation, thus aiding in tumor advancement [89].

A specific characteristic of metastasis is EMT, in which PKM2 contributes to the malignant invasion or metastatic traits of cancer cells by facilitating EMT. When PKM2 is transported to the nucleus, its activity is inhibited [90]. In the nucleus, PKM2 binds to transforming growth factor-beta-induced factor 2 (TGIF2) and brings histone deacetylase 3 (HDAC3) to the promoter of CDH1 gene, which results in a reduction in histone H3 acetylation and CDH1 gene expression encoding E-cadherin to cause transcriptional suppression of E-cadherin and EMT. These changes alter the adhesive properties of epithelial cells, promoting cell invasion and metastasis [90,91]. Mild inflammation stimulates inducible nitric oxide synthase (iNOS) to produce physiological levels of NO to promote glycolysis and induce cancer cell proliferation while enhancing resistance to oxidative stress. NO promotes the nuclear translocation of PKM2 via the EGFR/ERK2 pathway, which positively regulates glycolysis [92].

PKM2 holds a vital function within the nuclear compartment of cells, and interactions between proteins in the nucleus can be targeted at the molecular scale to address tumor development and progression.

#### 4.3.2. Mitochondrial PKM2 Inhibits Apoptosis, Regulates Mitochondrial Dynamics, and Drives Tumor Cell Survival

**Figure 5:**
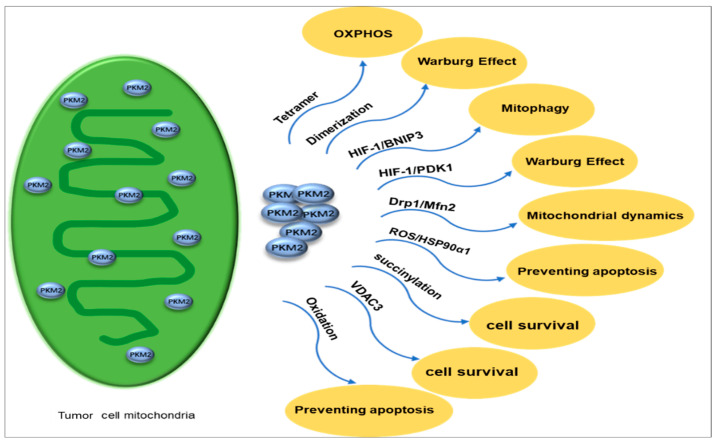
PKM2 exists in mitochondria in different proteoforms, with tetramers supporting OXPHOS and dimers favoring the Warburg effect. It activates HIF-1 to induce PDK1 and BNIP3, which inhibit OXPHOS and promote mitophagy, respectively. Altered mitochondrial dynamics occur through Drp1 downregulation and Mfn2 activation, leading to dysregulated fission and fusion. Under oxidative stress, PKM2 interacts with HSP90α1 to prevent apoptosis. Succinylated PKM2 and increased VDAC3 enhance mitochondrial functions, ensuring cell survival during nutrient deprivation. Oxidation of PKM2 at C358 and C424 enhances the glycolytic pathway’s ability to generate sufficient reducing power for ROS detoxification and reduce cell apoptosis. PKM2: Pyruvate kinases M2. OXPHOS: oxidative phosphorylation. PDK1: pyruvate dehydrogenase kinase 1. BNIP3: Bcl2/adenovirus E1B 19 kDa protein-interacting protein 3.

Mitochondria produce most ATPs through OXPHOS to provide energy for cells [93]. Previously, our research group used iTRAQ quantitative mitochondrial proteomics to investigate molecular alterations in energy metabolism pathways, specifically in OC, and effectively identified proteins such as PKM2 in the glycolytic pathway that are up-regulated in EOC [94]. The tetrameric proteoform of mitochondrial PKM2 has high catalytic activities to catalyze the pyruvate production from PEP, thereby enhancing the glucose-derived carbon flux towards OXPHOS [95]. In contrast, the dimeric proteoform of PKM2 represents a low-activity state for PKM2 and can promote glycolytic intermediates in the aerobic glycolysis pathway [96]. PKM2 leads to a decrease in intermediate products of the tricarboxylic acid cycle (TCA). PKM2 activates the HIF-1 transcription to activate pyruvate dehydrogenase kinase 1 (PDK1) [97,98,99] and interact with Bcl2/adenovirus E1B 19 kDa protein-interacting protein 3 (BNIP3) [98,99,100]. PDK1 inhibits pyruvate dehydrogenase (PDH) in the TCA cyle, which prevents the conversion of pyruvate to acetyl-CoA [101]. During hypoxia, BNIP3-induced autophagy is a survival mechanism of tumor cells to promote cancer progression [102,103] and decrease mitochondrial-encoded protein expressions in the OXPHOS pathway [100,102]. PKM2 induces AMP-activated protein kinase (AMPK) phosphorylation using mitochondrial activator AMP, which results in a decrease in mitochondrial activities [104,105,106]. Additionally, PKM2 induces mitophagy through the AMPK-mTOR signaling pathway [107,108]. Thereby, PKM2 is a promising tumor diagnostic biomarker because the dimeric proteoform of PKM2 redirects a glucose metabolism shift from mitochondrial OXPHOS to aerobic glycolysis (Figure 5).

Mitochondrial dynamics refers to the dynamic equilibrium state of fusion and fission in mitochondria [109]. Mitochondria adapt to cellular physiological needs and changes in the external environment by continuously undergoing division (fission) and fusion, participating in multiple cellular biological processes, including apoptosis, proliferation, migration, and energy metabolism [110,111,112,113]. Disrupted mitochondrial dynamics exist in tumors and are intricately linked to OC [114]. PKM2 overexpression regulates mitochondrial dynamics [115]. Under hypoxic conditions, mitochondrial fission caused by reactive oxygen species (ROS) leads to cisplatin resistance in OC cells [116], whereas PKM2 may translocate into mitochondria to inhibit mitochondrial division by reducing the expression of Drp1 [117]. Mfn2-mediated mitochondrial fusion can reduce ROS levels, promote autophagy, and inhibit OC progression through the AMPK/mTOR/ERK signaling pathway [118], whereas PKM2 can promote mitochondrial fusion by activating Mfn2 expression [119,120].

Mitochondria are the main intracellular source of reactive oxygen species (ROS) [121]. In various types of cancer, it has been observed that when cells are under oxidative stress or high levels of ROS, PKM2 is found to translocate to the mitochondria [122]. Enhanced metabolic activity, disrupted cell signaling, mitochondrial impairment, increased peroxisomal enzyme function, activation of oncogenes, or heightened activities of oxidases, purine nucleotide phosphorylase, lipoxygenase, and cyclooxygenase may lead to elevated levels of ROS within tumor tissues [123]. Oxidative stress has harmful effects on cells and may result in cell death [124]. Therefore, cancer cells enhance their inherent antioxidant capacity through specific mechanisms that preserve ROS balance and inhibit cell death.

Recently, PKM2 has been found to be the key regulator of cellular oxidative stress. Previous studies demonstrated its ability to activate the clearance system to deal with cell toxicity caused by elevated levels of intracellular ROS [125]. In the presence of ROS, PKM2 prevents apoptosis by interacting with its partner protein, HSP90α1. This is achieved by phosphorylation at residue Thr69 in Bcl2, which prevents cul3-based E3 ligase to be bond to Bcl2, stabilizes the Bcl2 molecule, and effectively inhibits cell apoptosis [123]. The mitochondrial function preservation depends on the interaction of PKM2 with Bcl2 on the OMM, which inhibits ROS release and prevents cell apoptosis triggered by oxidative stress [123].

In addition, the increased intracellular ROS levels cause the oxidation at residues C358 and C424 in PKM2, thereby inhibiting its PK activities, which further redirects the glucose flux towards the pentose phosphate pathway to provide sufficient reducing power for ROS detoxification [126,127]. In addition, PKM2 plays a role in nutrient stress. For example, glucose starvation can result in PKM2 succinylation, which promotes the mitochondrial translocation of PKM2 to function for the transition from proliferation to survival mode in cells [128]. PKM2 in mitochondria inhibits the VDAC3 (voltage-dependent anion channel 3) degradation through the ubiquitination/proteasome pathway, thereby increasing mitochondrial permeability to enhance ATP production and ensure cell survival under nutrient-deprived conditions [128].

#### 4.3.3. PKM2 Reshapes the Tumor Microenvironment in Extracellular Fluid

A solid tumor is mainly composed of tumor cells and the TME. The TME is highly heterogeneous and contains multiple cell types, such as fibroblasts, immune cells, platelets, adipocytes, and endothelial cells. Its soluble components, such as cytokines, chemokines, growth factors, metabolites, exosomes, and enzymes, play a crucial role in tumor growth, metastasis, and immune escape. PKM2, as a key metabolic enzyme in TME, enters the soluble environment through secretion, regulates the dynamic balance of TME, affects the expression of cytokines and growth factors, and then promotes the proliferation, invasion, and angiogenesis of tumor cells. Additionally, it includes a non-cellular component called the extracellular matrix (ECM), which is formed by polymer proteins and auxiliary molecules [129]. Extracellular PKM2 or secreted PKM2 is present in a patient’s blood in different types of cancers [130]. Research has found that PKM2 is secreted by cancer cells and enhances tumor cell migration via the Wnt/β-catenin and PI3K/Akt pathways [131]. The study also found that extracellular PKM2 activates the EGFR pathway in a self-secretory manner, promoting tumor cell growth and inducing EGFR phosphorylation [132]. Angiogenesis is a key indicator of tumor progression; a study found that the dimeric proteoform of PKM2 in the blood circulation promoted tumor angiogenesis by enhancing endothelial cell proliferation, migration, and adhesion to the ECM [133]. PKM2 released by neutrophils has been shown to contribute to tissue repair and wound recovery by facilitating angiogenesis [134].

**Figure 6:**
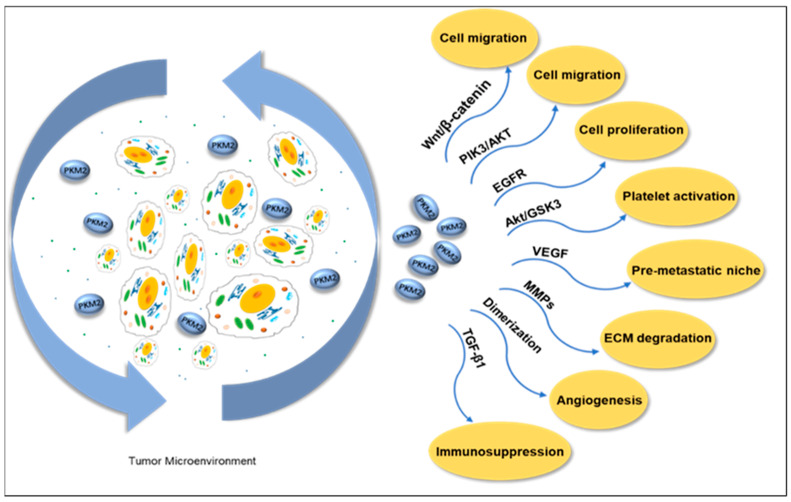
Extracellular PKM2 promotes cell migration through the PI3K/Akt and Wnt/B-catenin pathways in the tumor microenvironment. Activating the EGFR pathway promotes tumor cell proliferation. The Akt/GSK3β pathway activates platelets, promotes VEGF release and angiogenesis, and forms the pre-metastatic niche of tumor cells. In addition, it promotes the release of TGF-β1 to reduce the sensitivity of tumors to the immune system. It promotes cell migration by mediating the expression of MMPs and degradation of ECM. The dimeric PKM2 proteoform promotes tumor angiogenesis. PKM2: pyruvate kinases M2. EGFR: epithelial growth factor receptor. VEGF: vascular endothelial growth factor. TGF-β1: transforming growth factor beta 1. MMPs: matrix metalloproteinases. ECM: extracellular matrix.

Matrix metalloproteinases (MMPs) participate in the degradation of extracellular matrix (ECM), promoting the migration of tumor cells by degrading matrix components such as collagen and gelatin. After studying the effect of PKM2 expression on cancer cells at the molecular level, we found a correlation between PKM2, MMP2, and MMP9 expression. Western blot results demonstrated that PKM2 inhibition significantly reduced the expressions of MMP2 and MMP9. In addition, PKM2 knockdown inhibits tumor cell invasion ability [135]. PKM2 secreted by liver cancer exosomes triggers metabolic reprogramming in monocytes and induces phosphorylation of STAT3 within the cell nucleus, further upregulating differentiation-related transcription factors, thereby promoting monocyte differentiation into macrophages and reshaping the TME (Figure 6) [136].

Metabolic regulation mediated by PKM2 plays a crucial role in platelet activation, whose mechanism involves the induction of phosphatidylinositol 3-kinase (PI3K)-mediated Akt/GSK3βpathway. When resting platelets are activated, their energy metabolism shifts to aerobic glycolysis [137]. The activation of platelets can promote cancer cell proliferation and induce EMT, thereby causing tumor cells to detach and enter the circulatory system, enhancing the migration and invasion ability of cancer cells. Platelet activation is accompanied by the release of TGF-β1 and VEGF. TGF-β1 serves as a driving factor for EMT and functions as an important immunosuppressant, reducing tumor sensitivity to the immune system by inhibiting the differentiation of cytotoxic T lymphocytes (CTLs) and increasing the production of regulatory T lymphocytes (Treg) [138,139]. Platelet-derived VEGF promotes pre-metastatic niche formation and stimulates angiogenesis [140,141]. The inhibitors of PKM2, SHK [142], and quercetin (Que) [143] were co-encapsulated in platelet membrane-liposomes (PM-Lipo) for the treatment of tumor tissues. The results showed that this treatment significantly reduced the levels of TGF-β1 and VEGF inside platelets, effectively inhibited tumor cell proliferation and platelet activation, and blocked the interaction between platelets and tumor cells, thereby successfully suppressing the migration, invasion, and EMT processes [144].

### 4.4. Post-Translational Modifications of PKM

#### 4.4.1. Phosphorylation

Protein phosphorylation serves as a pivotal mechanism for signal transduction, wherein protein kinases catalyze the transfer of ATP and GTP γ-phosphate groups to amino acid residues on substrate proteins. Phosphorylation occurs at cytoplasmic and nuclear proteins, and amino acid residues serine, threonine, and tyrosine are the predominant phosphorylation residues in eukaryotic [145]. These residues are susceptible to phosphorylation in PKM2 and are closely associated with tumorigenesis and tumor progression. To correlate the phosphoproteomic dataset of cancer cell lines with the representative expression of the PKM2 proteoform in the TCGA database, OVCAR-3, MCF-7, and Jurkat cancer cell lines were specifically chosen for phosphoproteomic analysis to determine the phosphorylation status of PKM2. The findings revealed that PKM2 is expressed across different cell types, with PKM2pS37 being identified as the most significant phosphorylation site [21]. In triple-negative breast cancer (TNBC) cells, phosphorylation of PKM2 at the S37 site is associated with the cyclin-dependent kinase (CDK) pathway. The phosphorylation of PKM2pS37 is closely associated with the invasiveness of breast cancer, impacting the subcellular localization of PKM2 and exhibiting a negative correlation with overall survival and progression-free survival rates. These findings demonstrate that PKM2-pS37 phosphorylation might be a potential biomarker for breast cancer. Furthermore, researchers have developed a highly specific antibody against PKM2pS37 phosphorylation and validated its accuracy in detecting and localizing this modification through immunoblotting and immunofluorescence assays. This provides novel insights into the targeted therapy under the 3P framework [21]. Direct phosphorylation at residue Tyr105 in PKM2 inhibits the PKM2 tetramer formation, leading to a reduction in pyruvate kinase activity and an increase in dimerization, thereby enhancing protein kinase activity [146].

A study prospectively collected a total of 83 HGSC tissue samples and 20 normal control samples (fallopian tube, FT) and conducted mass spectrometry-based proteomics and phosphoproteomics to assess the differences in phosphopeptide abundance and protein abundance between the two groups. The results showed that, compared to normal tissues, tumors generally have increased protein phosphorylation levels and activated pathways related to proliferation, such as aurora kinase A (AURKA) and CDK-RB (retinoblastoma protein), which can be targeted for treatment using inhibitors approved by the US FDA, providing the rational basis to use these therapeutic methods in OC [147]. Research has found that depletion of KBTBD8 leads to the phosphorylation of Erk1/2 (p-Erk1/2) and Aurora A (p-Aurora A), resulting in a decrease in PKM1 expression [66]. The investigation of T cell acute lymphoblastic leukemia (T-ALL) has discovered that phosphorylation of PKM2 by cyclin D3-CDK6 dependency hinders PKM2 enzyme activity and facilitates cancer cell proliferation through inhibition of tetramer formation [148].

#### 4.4.2. Ubiquitination

Ubiquitin, a peptide consisting of 76 amino acid residues, is ubiquitously present in eukaryotic cells. Through the action of a series of enzymes, one or more ubiquitin molecules can be covalently attached to protein substrates, resulting in ubiquitination modifications [149]. This PTM can regulate protein expression levels and participate in various biological processes. Parkin, an E3 ubiquitin ligase, exhibits the ability to ubiquitinate lysine residues at positions 186 and 206 of PKM2, thereby reducing its pyruvate kinase activity. This inhibition contributes to the suppression of diseases such as lung cancer and Parkinson’s by attenuating glycolysis.

Deubiquitination also holds significance for PKM2; both otubain2 (OTUB2), a deubiquitinase with deubiquitinating activity, and proteasome non-ATPase regulatory subunit 14 (PSMD14) mediate the deubiquitination modification of PKM2. OTUB2 directly interacts with PKM2 to inhibit its ubiquitination by disrupting the interaction between PKM2 and E3 ubiquitin ligase Parkin, thus enhancing its pyruvate kinase activity and promoting the Warburg effect [150]. On the other hand, PSMD14 reduces PKM2-K63 (PKM2’s K63 chain) ubiquitination, which leads to a transition from the tetrameric form to the dimeric form of PKM2. Consequently, this weakens its pyruvate kinase activity while inducing nuclear translocation and further enhancing downstream oncogene transcriptional activation. These events promote metabolic reprogramming and cancer cell proliferation, ultimately driving malignant progression in OC [151]. In summary, both ubiquitination and deubiquitination mechanisms play pivotal roles in tumorigenesis and tumor development by regulating the activities of pyruvate kinase and protein kinase within PKM2; however, distinct regulatory mechanisms are often observed due to variations in modification sites or tumor types.

#### 4.4.3. Acetylation

Protein acetylation is to covalently attach an acetyl group (such as acetyl-CoA) to the lysine residues or N-terminus of a substrate protein, which is catalyzed by histone acetyltransferases (HATs/KATs). Initially, protein acetylation was found to mainly occur on histones in the cell nucleus. However, with advancements in mass spectrometry technology, we have discovered that non-histone lysine acetylation modifications are significantly present in the cytoplasm or other cellular organelles. Protein acetylation is closely associated with different cellular processes, including DNA damage repair, gene transcription, signal transduction, cell division, protein folding, metabolism, and autophagy. Moreover, protein acetylation regulates enzymatic activity, protein stability, crosstalk with other PTMs, subcellular localization, and protein/protein and protein/DNA interactions. PKM2 is a metabolic enzyme that can undergo multi-site acetylation, and its acetylation contributes to the occurrence and development of different tumors. The activities and stability of PKM2 protein are regulated by lysine acetylation. High glucose stimulation increases the interaction between acetyltransferase PCAF and PKM2, thereby promoting the acetylation at residue K305 in PKM2 to inhibit its activities. Additionally, PKM2- K305 acetylation promotes its binding with heat shock protein 70 (HSP70) and induces lysosome-dependent degradation through the chaperone-mediated autophagy (CMA) pathway. This process leads to a decrease in PKM2 activity, accumulation of glycolytic intermediates, and stimulation of amino acid biosynthesis, pentose phosphate pathway, and nucleotide synthesis metabolism, ultimately driving tumor cell growth [152,153]. The ectopic expressions of the PKM2 acetylation can mimic the mutant from K305 to Q305, which once again confirms that the accumulation of glycolytic intermediates promotes the proliferation and growth of tumor cells [152]. The accumulation of intermediate products in glycolysis affects the TME, which thereby alters drug sensitivity and leads to platinum resistance [154]. The PKM2-K433 site can be acetylated by the acetyltransferase p300, which further interferes with the binding of PKM2 to Fru-1,6-BP, which inhibits PKM2 activation and suppresses PKM2 dimer formation. This induces nuclear localization of PKM2, enhances protein kinase activity, and promotes breast cancer cell proliferation [155]. Silent information regulator factor 6 (SIRT6) is a Sirtuin family member of deacetylases, which mediates PKM2-K433 deacetylation to prevent nuclear translocation of PKM2 and inhibits its pro-cancer effect through acetylation in non-small cell lung cancer [156]. The testis-specific protein TSP50 can bind to PKM2, promote PKM2-K433 acetylation, reduce the activity of PKM2, promote the Warburg effect, and facilitate liver cancer cell proliferation [157]. Acetylated PKM2 acts as a coactivator in the cell nucleus, which binds to β-catenin to activate the Wnt/β-catenin pathway, enhancing the invasive, migratory abilities of a tumor cell. It is also closely associated with tumor progression and drug resistance in various types of cancer [158,159].

#### 4.4.4. Methylation

Protein methylation refers to the process in which a methyl group is transferred from S-adenosylmethionine to the corresponding protein under the catalysis of methyltransferases. Protein methylation mainly occurs on the amino groups of lysine or arginine side chains. The analysis of TCGA transcriptomic data and exon array data found that PKM gene intron 1 exhibits low methylation in multiple types of cancers [160]. The coactivator-associated arginine methyltransferase 1 (CARM1) can catalyze the methylation at sites R455, R447, and R445 in PKM2, which enhances PKM2 activities. This methylation also promotes breast cancer cell proliferation and migration [161]. Methylation of PKM2 at R445 and R447 promotes PKM2 tetramer formation and enhances its pyruvate kinase activities [162].

#### 4.4.5. Succinylation

Similar to acetylation, succinylation is a major protein modification that occurs on lysine residues. The enzymes involved in regulating protein succinylation and desuccinylation are mainly protein acetyltransferases and deacetylases [163]. Compared to methylation and acetylation, lysine succinylation modification can induce more changes in protein characteristics [164]. PKM2 succinylation plays an important role in the survival of tumor cells. PKM2-K311 succinylation inhibits the pyruvate kinase activities of PKM2 while promoting its protein kinase activity and playing an important regulatory role in the transcription of cellular inflammatory factors. In addition, we also found that TEPP46, a pyruvate kinase activator for PKM2, can reverse this process [165]. PKM2-K498 succinylation enhances the pyruvate kinase activities of PKM2, which thereby affects lung cancer A549 cell proliferation through regulation of the redox process. Meanwhile, SIRT5 can catalyze the desuccinylation of the succinylated PKM2-K498 [166]. Succinylation of PKM2 K498 caused PKM2 to transition from tetramer to dimer while also promoting nuclear translocation and enhancing PKM2’s protein kinase activity [165]. Succinylation at different sites of PKM2 can exhibit opposite regulatory effects on its kinase activity. Additionally, PKM2 succinylation can also promote its mitochondrial translocation [128], indicating the diverse functionality of PKM2 succinylation.

#### 4.4.6. Glycosylation

Protein glycosylation modification is the process in which sugar molecules are added to protein amino acid residues through the action of glycosyltransferases, forming glycosidic bonds and ultimately producing functional glycoproteins. The most common glycosylation of PKM2 is O-linked N-acetylglucosamine (O-GlcNAc). O-GlcNAc glycosylation involves two main enzymes: oxygen-linked N-acetylglucosamine transferase (OGT) and oxygen-linked N-acetylglucosaminidase (OGA) were detected. OGT is responsible for adding N-acetylglucosamine (GlcNAc) to the serine or threonine residues of proteins, while OGA is responsible for removing these sugar groups [167]. Glycosylation regulates various crucial biological processes, including apoptosis and cell cycle, by affecting protein stability, subcellular localization, activity, etc., thereby controlling physiological and pathological processes in organisms. PKM2 O-glycosylation inhibits PKM2 activities in HeLa cells by blocking its tetramerization, thereby promoting the aerobic glycolysis and growth of tumor cells. Collision-induced dissociation (CID)-based mass spectrometry analysis found that the glycosylation occurred at sites S362 and T365 in PKM2 [167]. O-glycosylation at sites T405 and S406 in PKM2 also causes the transition of PKM2 from tetrameric to dimeric form, which results in PKM2 nuclear translocation and inhibits its pyruvate kinase activity, effectively promoting the development of cancer [168]. A study found that epidermal growth factor (EGF) promotes the binding between OGT and PKM2, thereby facilitating PKM2 O-glycosylation [169], which is regulated by its acetylation and glucose levels. Under high glucose conditions, OGA can act as an acetyltransferase and induce acetylation of PKM2, thereby enhancing its O-glycosylation modification level. Furthermore, acetylated PKM2 can reduce tetramer formation and decrease pyruvate kinase activity, playing a crucial role in initiating tumor cell metabolic reprogramming and promoting cancer cell proliferation [167].

#### 4.4.7. Other Modifications

The lactylation of lysine 62 on PKM2 can enhance its kinase activity and facilitate the transformation of pro-inflammatory macrophages into a reparative phenotype by reducing PKM2 nuclear translocation and the transition from tetramers to dimers [170]. The small ubiquitin-like modification of PKM2 promotes human cancer cell aerobic glycolysis and proliferation [171]. A study found that under different levels of formaldehyde (FA) exposure, both tumor cells and normal tissue cells had an increasing trend in PKM2, thereby promoting the generation of the Warburg effect [172]. Follicle-stimulating hormone (FSH), Akt2, and AXL directly regulate PKM2 expression to stimulate OC cell proliferation, migration, invasion, and resistance to cisplatin and further promote aerobic glycolysis [22,173,174]. Meanwhile, PKM2 overexpression also regulates the expressions of multiple tumor-associated genes, for example, CCND1 upregulation and CDKN1A downregulation. This change is explained by promoting cell proliferation and survival in the S phase, providing new insights into the PKM2 biological roles in OC pathogenic mechanism [175].

## 5. Anti-Tumor Activity of Pyruvate Kinase Inhibitors

PKM2 inhibitors 3K and SIRT inhibitor MHY2245 effectively inhibit the expressions of PKM2 and disrupt glycolysis via modulating the Akt/AMPK/mTOR signaling pathway, which induces G2/M cell cycle arrest and autophagy, and inhibits colony formation ability and proliferation of cancer cells [176,177]. The PKM2 inhibitor SHK activates ATM/γH2AX to decrease the expression of PKM2 and trigger DNA oxidative damage and cellular apoptosis. The combination of SHK with olaparib can suppress homologous recombination repair, effectively hindering DNA recombinational repair of cancer cells. This demonstrates a synergistic anti-tumor activity [178]. These discoveries offer a viable approach for increasing the effectiveness of PARP inhibitors in the maintenance therapy of advanced OC patients.

## 6. Conclusions and Expert Recommendations in the Context of the 3PM Approach

**Figure 7:**
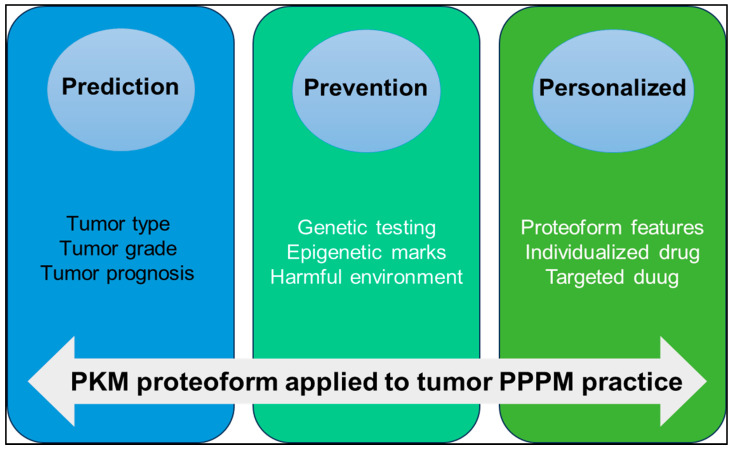
The application of PKM proteoform to predictive, preventive, and personalized medicine (PPPM) practice.

PKM is a key molecule in tumor metabolic reprogramming. Under different pathophysiological conditions, PTMs, alternative splicing, conformational changes, binding partners, subcellular localization and other factors promote the formation of different PKM proteoforms to promote tumor development through their respective functions and pathways. The concept of the proteoform has greatly enriched the connotation of the proteome. The study on proteoforms represents a higher level in the field of proteomics. Proteoformics plays an increasingly important role in precision health and precision medicine. For example, predicting clinically useful precise targets for small molecule drugs or elucidating therapeutical protein proteoforms specific to different disease categories, and 2DE-LC/MS provides technical support for large-scale investigation of proteoforms. We strongly recommend that the PKM proteoformics study be applied to PPPM practice for OC in the following three aspects (Figure 7).

(i) Prediction of tumor type and progression: The expression level of PKM proteoform is different in different types and grades of OC. Quantitative proteomic analysis of OC tissues showed that PKM2 was upregulated in OC [20]. The Ualcan database showed that PKM was highly expressed in OC. Detecting the expression level of PKM proteoform in blood or tissue specimens can help to predict the type, grade, and prognosis of tumors.

(ii) Prevention of tumors: The activity of PKM in normal cells is affected by a variety of regulatory mechanisms, including gene mutations, epigenetic modifications, and the regulation of signaling pathways, resulting in different proteoforms. Through genetic testing, we can identify high-risk gene mutations associated with tumors, conduct regular early screening, and then take preventive measures. By studying epigenetic markers, early changes in tumorigenesis are identified, and intervention strategies are developed to block the impact of these epigenetic alterations on tumorigenesis. Reducing harmful environmental exposure, such as high glucose and formaldehyde [167,172], to regulate PKM2 expression and activity is also an important way to prevent cancer.

(iii) Personalized medicine treatment strategy: The presence of PKM proteoforms can be used as an important marker in personalized medicine. Proteoformics allows researchers to detect the PTMs of various proteins, including phosphorylation, acetylation, methylation, ubiquitination, etc. Based on these personalized data, doctors can make individualized treatment plans according to the characteristics of the patient’s protein group. Specific proteoforms can enhance or weaken the relationship with drug response. For example, acetylated PKM2 acts as a coactivator in the nucleus to promote tumor drug resistance [158,159]. Therefore, new drugs can be developed by targeting modification sites to regulate the activity of PKM2, thereby enhancing the sensitivity of tumors to chemotherapy drugs. For example, the PKM proteoform specific for platinum resistance in OC has been identified, and this proteoform is involved in the alternative splicing machinery. Through the development of drugs specifically targeting these splice variants, specific alternative splicing factors or alternative splicing events, anti-tumor recurrence effects can be achieved. The development of antibodies specific for PKM proteoform can be used for personalized diagnosis or treatment and help to develop individualized treatment plans according to the specific biomarker situation of patients [21].

In summary, proteoformics can detect, identify, and quantify proteoforms and changes in proteoforms. PKM proteoforms play an important role in the tumor PPPM framework, which can help predict the type and prognosis of tumors, prevent the occurrence of tumors [7], and guide the formulation of individualized treatment strategies. Future studies should focus on validating PKM2-based biomarkers in large patient cohorts.

## Data Availability

All data and materials are provided in this article, which can be made available publicly.

## Code Availability

All protein and gene accession codes can be available in the Swiss-Prot and Genbank databases.

## Abbreviations

AMPK	AMP-activated protein kinase
AQUA	absolute quantification
AURKA	Aurora kinase A
BNIP3	Bcl_2_/adenovirus E1B 19 kDa protein-interacting protein 3
CA125	carbohydrate antigen 125
CARM1	coactivator-associated arginine methyltransferase 1
CDK	cyclin-dependent kinase
CID	collision-induced dissociation
CMA	chaperone-mediated autophagy
CTLs	cytotoxic T lymphocytes
CZE	capillary zone electrophoresis
ECM	extracellular matrix
EGF	epidermal growth factor
EMT	mesenchymal transition
EOC	epithelial ovarian cancer
FDA	Food and Drug Administration
FSH	follicle-stimulating hormone
FT	fallopian tube
GlcNAc	N-acetylglucosamine
HATs	histone acetyltransferases
HDAC3	histone Deacetylase 3
HE4	human epididymis protein 4
HGSC	high-grade serous carcinoma
IDS	interval debulking surgery
LC	liquid chromatography
LHX9	transcription factor 9
MMPs	matrix metalloproteinases
M_r_	molecular weight
MS	mass spectrometry
NACT	neoadjuvant chemotherapy
OC	ovarian cancer
OGA	oxygen-linked N-acetylglucosaminidase
OGT	oxygen-linked N-acetylglucosamine transferase
OTUB2	otubain2
OXPHOS	oxidative phosphorylation
PCS	primary cytoreductive surgery
PDK1	pyruvate dehydrogenase kinase 1
PDH	pyruvate dehydrogenase
PEP	phosphoenolpyruvate
PHD3	prolyl hydroxylase 3
pI	isoelectric point
PK	pyruvate kinase
PM	precision medicine
PKM2	pyruvate kinase M2
PKL	pyruvate kinase L
PPPM/3PM	prediction, prevention, and personalized medicine
PSMD14	proteasome non-ATPase regulatory subunit 14
PTMs	post-translational modifications
SAICAR	succinylaminoimidazolecarboxamide ribose-5′-phosphate
TAM	tumor-associated macrophages
TCA	tricarboxylic acid cycle
TCGA	The Cancer Genome Atlas
TGIF2	transforming growth factor-beta induced factor 2
TME	tumor microenvironment
TNBC	triple-negative breast cancer
Treg	regulatory T lymphocytes
VDAC3	voltage-dependent anion channel 3
VM	vasculogenic mimicry
2DGE	two-dimensional gel electrophoresis
